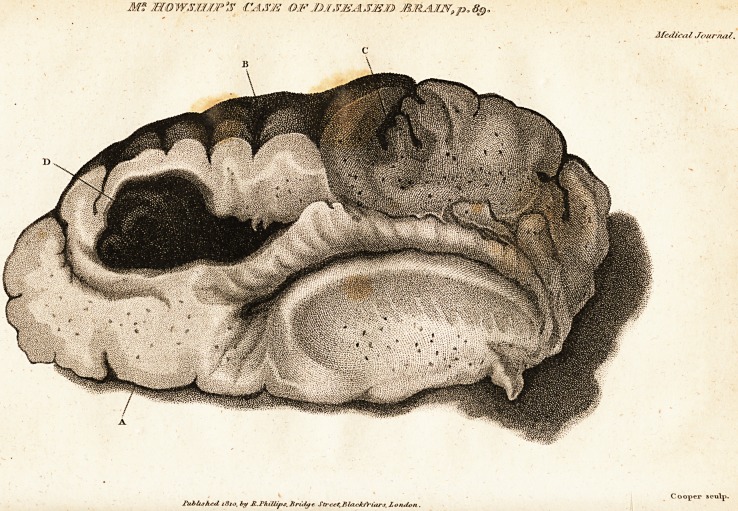# Observations on Diseases of the Brain, with Cases and Dissections

**Published:** 1810-08

**Authors:** J. Howship

**Affiliations:** Surgeon


### Medical and Physical Journal.

VOL. XXIV.] AUGUST, 1810. [NO. 138.

*Printed for R. PHILLIPS, by W. Thorne, Red Lion Court, Fleet Street, London.*

### OBSERVATIONS ON DISEASES OF THE BRAIN, WITH CASES AND DISSECTIONS.

BY MR. J. HOWSHIP, SURGEON.

### *Case of Extravasation of Blood upon the Surface and within the Medullary Substance of the Brain; the latter probably accidental, and productive of a fatal Termination.*

[With an Engraving.]

A. G. a young woman, about 22 years of age, had been for at least a year and a half, a great sufferer from rheumatic complaints, principally in the head. These were attended with a violent head-ach of so great intensity, as sometimes to produce delirium. In one attack, the pains in the head ran on to so alarming an extent, that a temporary state of general paralysis was the consequence, and she lost the use of her limbs. These complaints, however, were, after some time, conquered by the adoption of judicious measures, and within the space of two months she had recovered tolerably well all the powers of motion.

In the month of January, 1810, she left her friends, and came to London from a neighbouring town where she lived. The weather was at the time mild, but finding herself very unwell in the head, she loitered about the streets till the day was spent, and in the evening sat down at a door. About midnight she was desired to go away by a watchman; from this she excused herself by saying that "she could not." Very soon afterwards, however, she was obliged to get up, and with others, walked a considerable distance to the watch-house. Here she remained for the rest of the night, sitting on the cold stone floor. Her companions repeatedly remonstrated with her, endeavouring to persuade her to go and sit down by the fire, and to take something either to eat or drink, but to no purpose. All solicitations were useless; she declined, shook her head, and when urged to speak, said she did not want it. To those who were around her, it appeared that she was stupid from intoxication; yet, whenever much teased, she spoke, and that perfectly in reason. Once during the night she said something in reply to an inquiry what ailed her, and observed, that once before she had, from a fit of illness, lost the use of her limbs, and that from her present feelings, she feared that this would soon be the case again.

The following morning, with several other girls, she was taken before a magistrate, to give an account of herself. Those who then saw her could not help supposing her to be still under the influence of intoxication; but the persons who had spent the night with her, declared she had neither drank nor eaten any thing whatever in the night. As nothing satisfactory could be made out, she was brought back, and received into an infirmary, where she remained much in the same state, from the Tuesday night, when she was first brought into the watch-house, till the Saturday evening following, when, notwithstanding every exertion that had been made to save her, she died without any apparent struggle.

From the first she had continued in an uniform dull, silent, and stupid state, though not particularly disposed to sleep. When, with much persuasion, she was once prevailed on to take her medicine, she kept it on her stomach without difficulty.

She had always spoken rationally enough, but was observed to become more and more dull and inattentive to what passed around her, from the hour when she first came in to that of her dissolution.

EXAMINATION.

Upon opening the head, the first appearance of disease was found within the dura mater, which being raised and laid aside, exhibited the pia mater very much altered indeed from its natural state. The membrane was in every part highly vascular, both in the number and magnitude of its vessels. The most remarkable circumstance however, in the condition of the pia mater, was a degree of extravasation of blood, which might be traced all over the surface of almost every part of the brain. The extravasated fluid had formed superficial coagula, corresponding to the spaces between the convolutions of the brain.

This

This extravasation of blood appeared to have taken place very universally, not only from the capillary vessels of the pia mater, covering the external surface of each hemisphere, but also from those that form the tomentum cerebri, by dipping down between the circumvolutions of the brain; hence, upon making a section down through the substance of the cerebrum, several coagula of extrasated blood were cut asunder.

That particular extravasation however, which, in all probability, had been the immediate cause of death, was very distinct from the morbid appearances above described. Paring away the superior portion of the right anterior lobe cerebri, a coagulum was found deposited in the very central part of the medulla. In this situation, a vessel had ruptured and poured fourth a quantity of blood to the extent of nearly an ounce.

The engraving represents the coagulum that was discovered in the course of examination in the medullary part of the brain. Its precise size was not ascertained, as it was not disturbed from the cavity in which it was imbedded.

A. The inferior surface of the anterior lobe of the right hemisphere of the brain, where it lays immediately over the orbital cavity.

B. Points out one of those parts of the superior surface of the brain, where the pia mater was detached from its immediate contact with the convolutions, by the deposition of an angular stratum of coagulated blood. This appearance, from its being general upon every part of the surface of the brain was presumed to be the necessary consequence of severe rheumatic inflammation, when it attacks the membranes of the brain with so great a degree of violence, as was manifest in this case.

The circumstance of the extravasation having taken place only on those parts of the surface which mark the separation between the convolutions, may perhaps, be explained upon the principle of the circulating vessels having, in these parts, more freedom of action, and less support; whereas the vessels upon those portions of the membrane more immediately spread over the projecting ridges of the brain, meeting with more support, and to a certain degree even sustaining pressure, are less liable to yield.

This conjecture, however, affords no explanation why the same thing was found to have taken place in situations, where the substance of the brain lay in close contact with the pia mater, because the natural elasticity of the the structure of the brain is the same throughout the whole of its substance.

C. The appearance of the divided portion of a thin coagulated stratum of blood, thrown out from the vessels of the pia mater, dipping down between the convolutions into the substance of the brain.

D. The large deposition of blood scarcely coagulated, that was found in the medullary portion of the brain. From the circumstance of the large coagulum being yet in a gelatinous and almost fluid state, while the superficial coagula, connected with the pia mater, were all of a much more dense, firm, and dry texture; it seems almost more than probable, that the opinion given with regard to their date, is correct.

*Case of fatal Apoplexy, in which from the Rupture of Vessels, Blood was effused into the substance of the Medulla Oblongata.*

J. M———, an old man, aged 85, was apparently well in health on Sunday, February the 4th, 1810. In the night following he got out of bed to make water. A person who lay near him, being awake, enquired what he was about; to this he replied, that he only wanted to find the chamber pot. These words were no sooner out of his mouth, than he fell suddenly over the bedstead with his head to the floor, which he struck with great violence.

Assistance being obtained, he was raised up and laid in bed again, being in a state of insensibility, but breathing with much freedom, and laying in a state of perfect tranquillity, as if in a light repose.

The next day the pulse was full and hard, beating 92 in the minute; respiration was now rather heavy, and in some degree laborious; but there was no stertor. The eye-lids were still closed, and the jaw perceptibly falling.

On the day following (Tuesday) the respiration was attended with more labour and difficulty, as well as noise; the jaw more fallen, and the pulse slower than it was, but still very full and hard. In the evening he expired.

EXAMINATION OF THE BODY.

The head being opened, the vessels of the pia mater were found in a very turgid state, but they did not appear to have given way any where. The dark colour of the cortical substance in this brain, when compared with the medullary parts, formed a more striking contrast than is often often seen. Upon examining attentively the section of the cerebrum, a peculiar appearance, certainly of some disease in the extreme capillary blood-vessels was observed. This appearance was universally found but in the cineritious structure only. It resembled very much the appearance of petechiæ, or flea-bites, on the skin; and from the apparent correspondence with the known situation and distribution of the arteries in the cortical substance of the brain, it was concluded that this appearance arose from some diseased alteration in the structure of the capillary arteries, by which a portion of the colouring matter of the blood, just sufficient to stain the circumference of the vessel, was allowed to escape without proceeding to a more sensible degree of extravasation.

This appearance was found in every part of the cortical substance of the cerebrum, but it was more apparent about the basis of the brain than elsewhere.

The lateral ventricle on the right side was of the natural form, and contained only the usual quantity of fluid. On the left side, however, the appearance was very different. The quantity of lymph deposited on this side was equal to between an ounce and a half and two ounces.

The horns of the ventricle had suffered considerable distension from this accumulation of fluid, the discharge of which was not, as on the other side, followed by a collapsed state of the cavity of the ventricle. The appearance after the fluid had run off, was that of a large, extended, vaulted passage, or tube, which to the eye, gave a bold and remarkable impression. This cavity still preserved its natural turns, each of the cornua was equally enlarged with the others, and the whole space retained the exact form into which the contained fluid had moulded it.

In the ventricle on each side the plexus choroides was pale, but little changed from its natural state, except that on both sides this vascular expansion contained several small soft tumours, plentifully supplied with blood-vessels.

Upon examining various sections of the cerebellum, no appearance whatever of extravasation was observed, so that whatever might be the nature of the appearance in the cortical substance of the cerebrum, that of the cerebellum had not undergone any similar change, but was in all respects of healthy structure.

Removing that portion of the cerebellum, which, from its situation, makes the posterior part of the fourth ventricle, several small coagula of blood were observed, laying on the opposite side, being on the posterior surface of the medulla oblongata.

In order to ascertain the precise extent of this extravasation, the medulla oblongata was first removed from its situation, and then divided by a vertical section, carried from behind, forward. This section exhibited several thin strata of blood, approaching to a state of coagulation.

The disposition of these portions of extravasated blood was remarkable. Many circumstances in the anatomy of the brain favour the idea of the medulla oblongata being made up of a congeries of longitudinal fibres. This appears evidently to be the case, when some portions of its surface are observed with attention. On this supposition, however, it is not easy to understand upon what principle the blood, in escaping from its vessels, should have been disposed, as in this case it was found. Where blood is poured out into any muscular structure, it is known to separate, but rarely, if ever, to rupture the fibres. Should it, on the other hand, be taken for granted, that the structure of the medulla is merely a soft elastic matter, formed without any particular or essential arrangement of its particles, even then effusion of blood into its substance, would, as happens above in the medulla of the brain, assume the form of a mass, more or less solid; but in the present instance, the several very thin strata of blood were all disposed in the same manner exactly, laying transversely to the longitudinal fibres of the medulla, supposing such fibres to exist.

The coagula were found deposited at some small distance apart, one above another, not in a correct line, but so that estimating the probable effect of the whole, it would be a sudden and complete suspension of the functions of the medulla, considering it as the medium of connection between the brain and the system of spinal nerves. The arteries of the brain were, in many situations, completely ossified.

Upon opening into the cavity of the thorax, the heart was found in a very diseased state. The tendinous margin round the insertion of each auricle was converted into a firm mass of bone. The two great coronary arteries of the heart were, from their origin, behind the valves of the aorta, almost as far down as the apex of the heart, completely ossified.

The semi-lunar valves, at the mouth of the aorta, were much enlarged, and distorted from their natural form by the deposition of ossific matter within the margin of their attachment attachment to the artery. The whole extent of the arch of the aorta was enlarged to double its natural size, and completely diseased. The inner membrane being deprived of its natural smooth structure by the progress of ossification, was advanced to a very rare extent.

On examination of the internal surface of this artery, many of the ossified masses were found raised into irregular projections, intruding into the cavity of the vessel. The general appearance, however, was that of uniform patches of ossific matter, deposited between the middle coat of the artery and the inner membrane. From the surface of several of the most irregularly formed ossifications, the internal lining of the artery had apparently been removed ; in all probability, this had arisen from the irritation of these matters exciting an increased action in the absorbents. Another obvious effect of irritation, produced by these masses of bone upon the coats of the artery, appeared on applying a gentle pressure to several of them, upon which, globules of pus, some of them large, were seen to start out beneath, and some of the scales were found to be by this means, almost quite detached from their situation. Assisted by a lively imagination, the fancy might here have pronounced, that the ever watchful genius, destined to preside over the welfare of the constitution, had directed the commencement of the ulcerative process, under the delusive hope, that by this means it was possible the disease might be removed.

[An engraving, shewing the appearance of the medulla oblongata will be given in a succeeding Number.]

### *A fatal Case of Apoplexy from Extravasation of Blood upon the Brain.*

A labouring woman, in the 56th year of her age, sustained the shock of an apoplectic stroke, which deprived her of her senses and memory for many hours ; but from this she eventually recovered the perfect use of her faculties, and was apparently restored to a very good state of health. This happened in the year 1804.

Five years afterward, in 1809, she had a second attack of the same formidable nature ; she was suddenly deprived of her senses, and fell down in a state of stupefaction. From this attack also she was partially recovered ; but although, after some time, her memory was restored, she never again enjoyed the free and perfect use of her limbs. On the contrary, she was never afterward able to direct her steps with any tolerable precision or even safety.

In

In December, 1809, a few months after the second attack, she was afflicted a third time. She had just reached the top of a stone stair case, when she instantaneously dropped down; and such was the ill fortune of the moment, that she fell to the bottom of the stairs. By this accident was received a very violent blow upon the right parietal bone, dividing the scalp, and exposing the bone to some extent. In a state of insensibility she was taken to an hospital, excessively restless, and constantly moaning. The hair being removed, the scalp was found very much bruised, with some degree of laceration. In order that the state of the bone might be more decidedly ascertained, the scalp was divided very freely by a crucial incision; from this it appeared that the skull was neither depressed nor fractured. The pupil of the eye was observed to be in a state of contraction, which was neither altered by the approach of a bright light, nor by leaving the apartment almost in darkness; the iris therefore was in a state of permanent contraction, as well as insensibility. The pulse was in a natural state, beating 76.

Sixteen ounces of blood were immediately taken from the arm, and a cathartic enema directed to be injected as soon as it could be got ready. Thirty drops of the tincture of opium were ordered to be given in a draught in the evening. This woman's condition, during the evening and night, was not that of apoplexy exactly, for she would frequently answer when spoken to, but still spoke incoherently, and always as if impatient from pain.

The following morning the account was very unpromising; there had been considerable hæmorrhage from the mouth, nose, and ears. The injection had returned, soon after being thrown up, without seeming to have produced much action in the bowels. In other respects she was much the same, being still quite incapable of answering rationally any question that was put to her. She seemed disposed to lay quiet as if asleep, yet would instantly open her eyes on being spoken to, but never apprehended what was said to her.

In this state she remained without taking either medicine, or support, that was of consequence, for five days, the pulse throughout remaining in its natural state, not hurried, nor slower than before, neither hardened, nor perceptibly oppressed. About the expiration of the fifth day she expired.

EXAMINATION AFTER DEATH.

The scalp being turned back, the cranium was found without without any trace of fracture. Upon raising the upper part of the skull an extensive mass of coagulated blood, of a firm, condensed, leathery consistence, was found deposited within the bone, and upon the dura mater. This mass, in extent as well as situation, corresponded very exactly with the temporal space upon the right side of the head, extending downward quite to the basis of the cranium. This blood had nothing of the rich scarlet and purple colour of blood recently coagulated, but was of a reddish brown or chocolate colour.

The dura mater being raised, and a division made at the anterior extremity of the falciform process, in order that the membrane might be with more freedom turned back, the whole of the surface of the left hemisphere of the brain and pia mater was discovered bathed in blood, partly fluid and partly grumous. The hemisphere on the opposite side was altogether free from this appearance. On the surface of the right hemisphere, the pia mater was in many places observed to be separated from the arachnoid membrane by the interposition of a serous fluid, not in any considerable quantity, but sufficient to show the prevailing tendency to lymphatic effusion.

The extravasation of blood was, on further examination, found to have universally diffused itself over every part of the cerebellum, as well as cerebrum, upon the left side. Perhaps the quantity of blood that had formed the more recent extravasation, might have been in the whole equal to three or four ounces.

On opening into the lateral ventricles, the plexus choroides bore the only trace of disease ; several little tumors resembling small hydatids were found in each plexus, towards that portion deposited in the descending horn of the ventricle.

The left side of the basis of the cranium could scarcely be seen, from the quantity of effused blood that remained after the lobes of the brain were removed.

The branches of the internal carotid were in several places found to be considerably ossified.

The medulla oblongata and the medulla spinalis were now taken from their cavity, and here the only peculiar appearance met with was in the form of the left vertebral artery. This vessel, at the point where it emerges from its canal in the occipital bone, had become considerably larger than usual, almost to double its proper diameter; but the alteration in texture was more remarkable than even that of its form, the artery being thinner than in its natural natural state, and of a rigid feel, like dried parchment.—The enlargement of this artery was observable in some degree as far as its junction with the vertebral artery on the opposite side.

It may in this case be reasonably conjectured, that although the third attack, and particularly the accidental fall which attended it, had proved fatal by the pouring out of the large quantity of blood that was found in a fresh and fluid state on examination of the head after death, yet the coagulum within the right temple was the consequence of the second attack. Under these circumstances we see then, the brain had been, from the deposition of this blood, in the first instance, overpowered, and its functions nearly extinguished; but that, assisted probably by the means adopted for its relief, the circulating volume of blood by slow degrees subsided into a smaller compass, so that even with this mass of blood intruding into the space formerly occupied by the brain alone, there was an almost perfect recovery of the faculties, although the decision and force of the voluntary powers were not re-established.

*Mill Street, Hanover Square, July 9, 1810.*

## Figures and Tables

**Figure f1:**